# Pressurized carbon dioxide as a potential tool for decellularization of pulmonary arteries for transplant purposes

**DOI:** 10.1038/s41598-020-60827-4

**Published:** 2020-03-04

**Authors:** Alicia Gil-Ramírez, Oskar Rosmark, Peter Spégel, Karl Swärd, Gunilla Westergren-Thorsson, Anna-Karin Larsson-Callerfelt, Irene Rodríguez-Meizoso

**Affiliations:** 1 Centre for Analysis and Synthesis, Department of Chemistry, Lund University, SE-22100 Lund, Sweden; 2 Lung Biology, Department of Experimental Medical Science, Faculty of Medicine, Lund University, SE-22184 Lund, Sweden; 3 Cellular Biomechanics, Department of Experimental Medical Science, Faculty of Medicine, Lund University, SE-22184 Lund, Sweden

**Keywords:** Analytical biochemistry, Lipids, Quality of life, Medical research

## Abstract

Vascular bio-scaffolds produced from decellularized tissue offer a promising material for treatment of several types of cardiovascular diseases. These materials have the potential to maintain the functional properties of the extracellular matrix (ECM), and allow for growth and remodeling *in vivo*. The most commonly used methods for decellularization are based on chemicals and enzymes combinations, which often damage the ECM and cause cytotoxic effects *in vivo*. Mild methods involving pressurized CO_2_-ethanol (EtOH)-based fluids, in a supercritical or near supercritical state, have been studied for decellularization of cardiovascular tissue, but results are controversial. Moreover, data are lacking on the amount and type of lipids remaining in the tissue. Here we show that pressurized CO_2_-EtOH-H_2_O fluids (average molar composition, *Χ*_CO2_ 0.91) yielded close to complete removal of lipids from porcine pulmonary arteries, including a notably decrease of pro-inflammatory fatty acids. Pressurized CO_2_-limonene fluids (*Χ*_CO2_ 0.88) and neat supercritical CO_2_ (scCO_2_) achieved the removal of 90% of triacylglycerides. Moreover, treatment of tissue with pressurized CO_2_-limonene followed by enzyme treatment, resulted in efficient DNA removal. The structure of elastic fibers was preserved after pressurized treatment, regardless solvent composition. In conclusion, pressurized CO_2_-ethanol fluids offer an efficient tool for delipidation in bio-scaffold production, while pressurized CO_2_-limonene fluids facilitate subsequent enzymatic removal of DNA.

## Introduction

Cardiovascular diseases (CVDs) are responsible for 17.9 million deaths per year in the world (31% of total deaths)^1^. In 2015, the global prevalence of arterial hypertension (AHT), the most prevalent risk factor for CVD development, was estimated to be around 30–45% of the adult population, increasing up to 60% in people above 60 years of age^2^. Moreover, the prevalence of AHT is estimated to increase by 15–20% in 2025^2^. Pulmonary arterial hypertension (PAH), a sub-form of AHT, is characterized by breakdown of elastic fibers and alterations in the cross-linking of collagen, resulting in remodeling the extracellular matrix (ECM) in pulmonary arteries^3,4^. Hypertrophic remodeling of the media and endothelial cell dysfunction result in a high vascular resistance and thrombosis^4,5^, potentially leading to right ventricular failure and death in severely affected patients.

Organ or tissue transplantation is the last option proposed for such CVDs-affected patients with a poor prognosis. However, the lack of compatible organs and tissues constitutes a major limitation. Even though the global rate of transplantation increased by 7.25% between 2015 and 2016, reaching a rate of 15.5 organs transplanted per hour^6^, less than 10% of the transplant needs are covered. Consequently, patients often have to wait long time for transplantation, resulting in worsening of their medical condition. Furthermore, those that are offered a transplantation require life-long immune therapy to reduce the risk for organ/tissue rejection^7^. Large efforts have been invested in approaches such as own-tissue regeneration, synthetic scaffold construction or bio-scaffold production to increase the availability of tissues for transplantation purposes^8^.

The potential of decellularized blood vessels as a source of vascular grafts have long been recognized, however ideal decellularization strategies are still sought after^9^. Decellularization of native tissue should reduce immunogenicity while preserving structure and biomechanical properties. Decellularized tissues are composed of natural ECM components and can be modified by repopulating cells allowing for remodeling, repair and growth *in vivo*. The material produced by decellularization of native tissues, termed bio-scaffold or bio-extracellular matrix (bio-ECM), are mainly composed of long-chain structural components, *e.g*. elastin and collagen^10^. These structures must be free from cellular components, but still maintain their native architectural and mechanical characteristics^11^. Lipid residues may hamper bio-ECM production as: (i) lipid bilayers constitute a barrier that hinders extraction of protein and genetic material; (ii) lipids, such as phosphatidylinositols, which are anchored to membrane proteins are involved in the development of immune responses^12,13^; (iii) some lipids hamper recellularization by impairing cell attachment^14^; and iv) certain lipids may act as a source of pro-inflammatory fatty acids, such as arachidonic acid^15^. Moreover, residual antigens (dsDNA) in the bio-ECM should be kept at a minimum to avoid activation of the immune system, which may lead to tissue rejection^16,17^. For a successful bio-ECM production, dsDNA and lipids (delipidation) need to be removed, while preserving ECM functionality. Ideally, the decellularization method should allow some bioactive molecules, *e.g*. growth factors and cell adhesion molecules, such as glycosaminoglycans, to remain in the ECM^11^.

Currently, the dominating techniques used for tissue decellularization utilize surfactant-based methodologies. Ionic, non-ionic or zwitterionic detergents, i.e. sodium dodecyl sulfate (SDS), Triton X-100 and CHAPS, respectively, results in efficient decellularization of the tissue, including removal of lipids^11,18–20^. Some decellularization protocols also include nucleases, which degrades genetic material in the tissue^18,21^. However, detergents often damage the ECM by disrupting the protein tertiary and quaternary structures^19,22^. Studies have revealed SDS to efficiently remove glycosaminoglycans, thereby causing collagen damage^11,22^. Detergents have also been shown to reduce levels of beneficial growth factors in some tissue^23^. Moreover, residual surfactants are cytotoxic^24^, yielding detrimental effects on the subsequent recellularization.

As an alternative to detergents, supercritical carbon dioxide (scCO_2_) has been proposed as a safer, non-toxic and non-residual technology to obtain bio-ECMs from multiple tissues, including porcine heart valve, equine tendon, porcine esophagus, bovine cartilage, human skin, porcine aorta, and human amniotic membrane^17,21,25–30^. The vast majority of these studies have revealed no or a very moderate influence of pressurized carbon dioxide on ECM structure and mechanical properties. However, while delipidation is crucial for the usefulness of the bio-scaffold, studies on ECM lipid composition before and after the treatment are lacking. Despite claims about the capacity for scCO_2_-cosolvents to remove lipids from biological tissue, only two of the studies mentioned above show results regarding lipid analysis. Sawada *et al*. (2008) showed a decrease in the content of total phospholipids in porcine aorta, quantified by enzymatic analysis^30^. Wehmeyer *et al*.^25^ showed no positive staining of membrane lipids in amniotic membrane, but the lipid alkyl chains were still present, according to results from differential scanning calorimetry.

Regarding DNA removal, studies remain controversial. Some authors claim successful results using scCO_2_ with the addition of ethanol or other modifiers (arguably forming a non-supercritical pressurized fluid)^21,27,30^. Other publications concluded that it was not possible with scCO_2_, even if aqueous or ethanol modifiers were used^17,29^. These discrepancies could result from differences between studies with respect to the origin, type, compactness, thickness and size of the tissue, as well as from differences in tissue pretreatment, composition and physical state of the fluid (supercritical *vs* non-supercritical pressurized fluids).

Notably, some of the published works claiming supercritical fluid decellularization are presumably not treating the sample with supercritical but rather one-phase pressurized fluids composed of CO_2_ and cosolvent. These fluids have higher polarizability than supercritical mixtures and better mass transfer properties than neat cosolvents^31^. They occur when excess of cosolvent is added to the scCO_2_, and specific pressure and temperature conditions are used. The physical state of the solvent mixture is crucial for the outcome of any extraction process and may be of most importance for the decellularization process in particular. A more appropriate use of nomenclature is advisable in the quest of decreasing the controversy found in the literature.

In this study, we examine the efficiency of one-phase pressurized CO_2_-cosolvent fluids and neat supercritical CO_2_ in the decellularization of porcine pulmonary arteries, paying special attention to lipid removal. Several treatment conditions (15.0–30.0 MPa, 90–120 min, 37–40 °C) were explored using a design of experiments (DoE) approach. The amount and identity of the lipids removed from pulmonary artery by pressurized CO_2_-cosolvent fluids was studied by ultrahigh performance supercritical fluid chromatography (UHPSFC) coupled to quadrupole time-of-flight mass spectrometry (QTOF-MS/MS) followed by statistical analysis. Two different methods were used to evaluate the removal of DNA from the tissue, i.e. DNA quantification and staining of nuclei. Finally, the integrity of the ECM after pressurized treatment was evaluated in terms of tissue hydration, matrix morphology and mechanical properties.

## Materials and Methods

### Biological material

**Figure 1:**
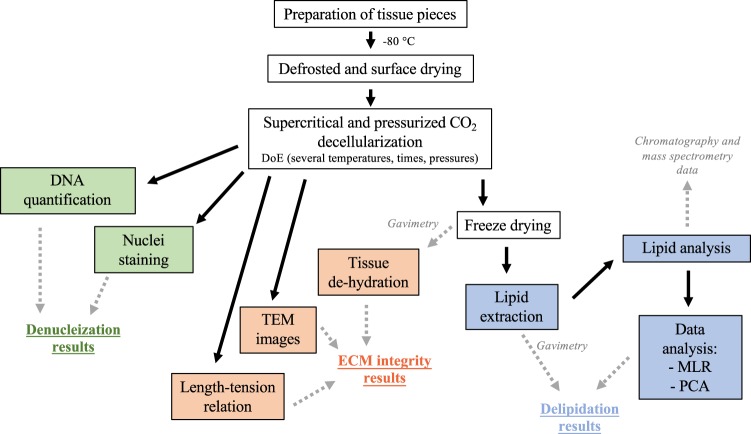
Workflow for decellularization of porcine pulmonary arteries. Multiple linear regression for DoE (MLR) and principal component analysis (PCA) were used as statistical techniques for data analysis.

Lungs from outbred pigs were provided by the Thoracic Surgery research group (Lund University, Sweden). Pulmonary arteries (PA) were excised, rinsed in PBS and cut into pieces of 5 mm^2^ size. PA sections (n = 250 from two porcine lungs) were randomized and kept at −80 °C until use. Immediately before treatment, samples were de-frosted at room temperature and the tissue dried for three seconds using absorbing paper (Whatman N°4) (Fig. 1). All methods were performed in accordance with the relevant guidelines and regulations. The study was approved by the local research ethical committee at Lund University (2015–174).

### Reagents and standards

Ultrapure carbon dioxide (99.9993% purity) was provided by AGA GAS AB (Växjö, Sweden). Ethanol (95% v/v) and limonene (97% purity), used as cosolvents for the pressurized treatment, were purchased from Solveco (Rosersberg, Sweden) and Sigma- Aldrich (St. Louis, MO), respectively. Formaldehyde 4% aqueous solution buffer (VWR, Leuven, Belgium) was used as histological tissue fixative. Ultrapure water (18 Ω/cm) was dispensed by Milli-Q devices from Merck Millipore (Darmstadt Germany). Methanol (LC-MS grade) and dichloromethane (stabilized with 0.002% methyl-2-butene) were provided by VWR Chemicals (Fontenay-sous-bois, France). Ammonium formate (≥99% purity), and the lipid standards glyceryl tripalmitate (TG 16:0/16:0/16:0; ≥99% purity), 1,2-dipalmitoyl-sn-glycero-3-phosphocholine (PC 16:0/16:0; ≥99% purity), sphingomyelin from chicken egg yolk (SM 18:1/16:0; ≥98%) and stearic acid (≥98.5% purity) were purchased from Sigma-Aldrich. Internal standards, N-oleoyl(d_9_)-D-erythro-sphingosylphosphorylcholine (SM 18:1-d_9_; ≥99% purity), 1-pentadecanoyl-2-oleoyl(d_7_)-sn-glycero-3-phosphocholine (DPPC, PC 15:0–18:1-d_7_; ≥99% purity), 1-oleoyl(d_7_)-rac-glycerol (MG 18:1-d_7_; ≥99% purity), 5Z,8Z,11Z,14Z-eicosatetraenoic-16,16,17,17,18,18,19,19,20,20,20-d_11_ acid (arachidonic acid d_11_ ≥99% purity) and 1,3-dipentadecanoyl-2-oleyol(d_7_)-glycerol (TG 15:0–18:1-d_7_-15:0; ≥99% purity) were from Avanti Polar Lipids Inc. (Alabaster, AL).

For histological stainings, Mayer’s Hematoxylin and 0.2% Eosin Y solution were acquired from Histolab (Gothenburg, Sweden) and for staining of elastic fibers a Modified Verhoeff Van Gieson Elastic Stain Kit (HT25A) from Sigma-Aldrich was purchased. For decellularization of reference tissue sodium deoxycholate (SDC, ICN Biomedicals Inc., Aurora, OH, USA) and sodium dodecyl sulfate (SDS, >98% purity, VWR, Radnor, PA, USA) was used.

For the preparation of TEM samples, xylene was purchased from Histolab (Gothenburg, Sweden), acetone 99.8% for Analysis Emsure® ACS from MilliporeSigma (Billerica, MA), Poly/Bed® 812 Embedding Media from Polysciences, Inc. (Warrington, PA), 4% Uranyl acetate from Agar Scientific (Stansted Essex, England, UK) and lead citrate from Merck (Darmstadt, Germany).

### Supercritical and pressurized carbon dioxide mediated decellularization

**Figure 2:**
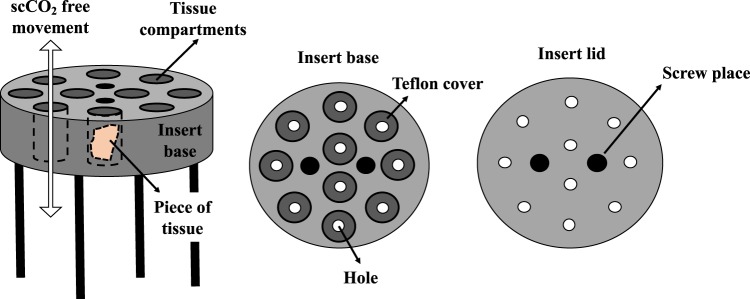
Details of the sample holder specially design for decellularization of pulmonary arteries. Ten pieces of tissue were loaded per decellularization condition.

Tissue pieces were individually loaded in a sample holder specially designed by Prototypverkstaden (Lund, Sweden) (Fig. 2). Samples were then treated using an in-house constructed equipment, as previously reported^32^. Briefly, the sample holder was placed in a stainless-steel extraction vessel with a volume of 80 mL (Ångström Laboratory, Uppsala University, Uppsala, Sweden), mounted on top of a magnetic stirrer (VWR, Leuven, Belgium) inside a GC-oven (HP 5890 GC, Hewlett-Packard Co., Palo Alto, CA). A thermocouple was used to control the temperature inside the vessel and a high-pressure syringe pump (Isco 260D, Teledyne Technologies Inc., NE) delivered liquid carbon dioxide from a dip-tube CO_2_ cylinder to the bottom of the vessel. Depressurization of the vessel was performed using an upper valve, which exclusively released CO_2_, and a lower valve, which released cosolvent. For safety reasons, a burst disk (maximum pressure of 40 MPa, Zook Enterprises, Sheffield, UK) was placed between the vessel and the upper CO_2_ vent valve.

The extraction vessel was preheated, followed by addition of 15 mL of ethanol:water (95:5, v/v) or limonene at the bottom of the vessel, when cosolvent was used. The water (5% in ethanol) was introduced to avoid tissue dehydration to the maximum possible extent, and it also helps to increase the polarizability of the mixture. Then, the vessel was closed, tubing connected, and oxygen purged by pumping CO_2_ for 1 min to avoid oxidation of the substrate. Subsequently, the system was pressurized to yield the desired pressure. During the decellularization process, the pump was set to maintain a constant pressure to compensate for undetectable leaks. After depressurization, cosolvent remaining in the vessel was removed by passing a constant flow of scCO_2_ rinse (8.5 MPa, 37 °C, 8 mL/min, 10 min). Samples destined for histological evaluation (n = 3) were immersed in 5 mL of 10% formalin for 12 h, rinsed with 10 mL of 70% EtOH for 30 min (x3) and then kept in 10 mL of 70% EtOH at 4 °C until examination (Fig. 1). Samples to be used for lipid analyses were freeze-dried and kept at −80 °C until extraction.

Two full-factorial design, one for each cosolvent, with three center points, were created in MODDE 10.1 (Sartorius Stedim Biotech, Malmö, Sweden) to investigate the impact of pressure (15.0–30.0 MPa), time (90–120 min) and temperature (35–40 °C) on tissue decellularization (Table 1). Higher pressures are obtained by adding more CO_2_ to the mixture, thus modifying the composition of the mixture. To simplify the DoE, we have investigated pressure as a variable while we have given composition a fictitious constant value, reflected as the average molar fraction.

**Table 1:** Details of the two full factorial designs used to evaluate the impact of pressure, temperature and time on supercritical CO_2_ mediated decellularization of pulmonary arteries.

Experiment	Pressurized CO_2_-EtOH-H_2_O (average *Χ*_CO2_ 0.91)			Pressurized CO_2_-limonene (averaged *Χ*_CO2_ 0.88)		
	Pressure (MPa)	Temperature (°C)	Time (min)	Pressure (MPa)	Temperature (°C)	Time (min)
1	30.0	35.0	90	15.0	35.0	90
2	22.5*	37.5	105	30.0	35.0	90
3	15.0	40.0	90	30.0	40.0	120
4	22.5*	37.5	105	15.0	35.0	90
5	22.5*	37.5	105	15.0	40.0	90
6	30.0	35.0	120	22.5*	37.5	105
7	15.0	40.0	120	15.0	40.0	120
8	30.0	40.0	90	30.0	40.0	90
9	15.0	35.0	90	22.5*	37.5	105
10	15.0	35.0	120	22.5*	37.5	105
11	30.0	40.0	120	30.0	35.0	120

*Central points.

Total amount of remaining lipids per lipid class (μg of lipid class per mg of freeze-dried tissue) and remaining DNA were used as response variables to estimate the delipidation and DNA removal efficacy, respectively. PA pieces were subjected to neat scCO_2_ treatment for 120 min at 30.0 MPa and 40 °C.

### Enzymatic DNA removal

Tissue samples subjected to neat scCO_2_ and pressurized CO_2_-cosolvent fluids were allowed to equilibrate in enzyme buffer (20 mM tris(hydroxymethyl)aminomethane, 20 mM NaCl, 2 mM MgCl_2_) for 30 min, followed by treatment with 90 U/mL of benzonase endonuclease for 30 min at 37 °C, 1 mL per 5 mm^2^ sample. Samples were subsequently washed three times in PBS during a total of 44 h, followed by fixation in formalin or quantification of residual DNA.

### Evaluation of nuclei removal

The nuclei remaining in the specimen after treatment were estimated by hematoxylin and eosin (H&E) staining and quantification of residual DNA.

#### Hematoxylin and eosin staining of treated pulmonary arteries from pigs

Formalin fixed samples were dehydrated, embedded in paraffin and sectioned with a thickness of 4 µm. Sections corresponding to the central portion of the sample were selected for H&E staining. Untreated tissue as well as tissue submitted to neat scCO_2_ were used as references.

#### DNA quantification

Residual double stranded DNA (dsDNA) were quantified by fluorescent nuclei acid staining using the Quant-iT^TM^ PicoGreen^TM^ dsDNA Assay Kit (Molecular Probes, Inc., Eugene, OR). Samples were lyophilized and homogenized using a Omni Tissue Homogenizer (Omni, Kennesaw, GA) followed by incubation with 200 U/mL of Proteinase K (Sigma-Aldrich) for 16 h at 37 °C. Samples were then centrifuged at 2000 × *g* for 10 min and dsDNA quantified in the supernatant (ng dsDNA/initial wet weight of the sample) according to manufacturer’s instructions.

Fresh tissue submitted to detergent based decellularization^33^ were used as positive controls. Briefly, pieces of PA were treated with a combination of 0.5% sodium deoxycholate (SDC) and 0.5% sodium dodecyl sulfate (SDS) for 24 h at room temperature with constant shaking. Samples were subsequently washed three times in phosphate buffered saline (PBS) for a total of 42 h, followed by fixation in formalin or quantification of residual DNA.

### Extraction of lipid residues from treated tissues

Lipids were recovered by a dichloromethane/methanol/water-based extraction method adapted for porcine pulmonary arteries^34^. Briefly, freeze-dried tissue pieces (see Supplementary Fig. S1) (in randomly assembled batches of 20 samples) were disrupted in a Qiagen TissueLyser (Qiagen GmbH, Hilden, Germany) for 10 min (1 min per cycle) at 25 Hz, followed by extraction of lipids as previously described in detail^34^. Extracts were dried under a stream of nitrogen gas, weighted for gravimetric analysis and stored at −80 °C until analysis.

### Lipid analysis

Lipid fractions (n = 4 per condition) were dissolved in 50 µL of CHCl_3_:MeOH (1:1, v/v) and analyzed on an Acquity Ultra Performance Convergence Chromatography (UPC^2^, Waters, MA, USA) system equipped with an Acquity UPC^2^ Torus DIOL column (130 Å, 1.7 µm, 3 mm ×100 mm, Waters, MA, USA) and fitted with a Van Guard Torus DIOL (130 Å, 1.7 µm, 2.1 mm ×5 mm, Waters, MA, USA) pre-column. Chromatography was performed as previously described in detail^34^, with a few modifications. The cosolvent (methanol containing 10 mM of ammonium formate) gradient was: 0 min, 2%; 2 min, 2%; 4 min 13%; 7 min, 27%; 7.5 min, 35%; 8.5 min, 35%; 9 min 2%; 11 min 2%. The flow rate was 1.6 mL/min.

Mass spectrometric detection was performed using a Xevo-2G quadrupole time-of-flight mass spectrometer (QTOF-MS; Waters, MA, USA). Make-up solvent (10 mM ammonium formate in methanol) was supplied at 0.25 mL/min and back-pressure regulated using two T-pieces, placed between the chromatographic system and the mass spectrometer (split ratio about 1:100)^34,35^. The capillary voltage was set at 3.0 kV and 2.5 kV for positive and negative electrospray ionization mode, respectively. The sampling cone voltage was set at 40 V, the cone gas flow rate at 100 L/h and the drying gas flow rate at 800 L/h, with a source and drying temperature of 120 °C and 200 °C respectively. The mass spectrometer was operated in MS^E^ mode with a scanning range of *m/z* 150−1000, with a resolution of at least 20000 at *m/z* 500–900 using leucine-enkephalin (1500 ng/mL at 5 µL/min) for internal calibration. Data were processed in MassLynx v4.1(Waters, MA, USA) and Mzmine2^36^.

Lipids were identified by exact mass using LipidMaps® Lipidomics gateway (San Diego, CA) and published data^34,37^ retention times and by fragments.

Absolute quantification was performed using an external calibration curve. Calibrant mixtures were composed by tripalmitin, DPPC, sphingomyelin and stearic acid in CHCl_3_:MeOH (1:1, v/v) with concentrations ranging from 10 to 50 ppm. Samples, blanks and calibration mixtures (50 µL) were spiked with 17 µL of the internal standard (IS) stock solution, which was composed of a mix of TG 15:0–18:1-d_7_-15:0, MG 18:1-d_7_, PC 15:0–18:1-d_7_, SM 18:1-d_9_ and arachidonic-d_11_ acid at 200 ppm in CHCl_3_:MeOH (1:1, v/v). The final concentration of each IS in samples and calibrant mixtures was 50.7 ppm.

Limit of detection (LOD) and limit of quantification (LOQ) were defined as three and ten times the signal-to-noise-ratio (S/N), respectively, for the analyte/internal standard-ratio and were calculated separately per lipid species (n = 3 per lipid species). Lipid species with levels below their respective LOD were considered absent and those with levels below LOQ were not quantified and hence not used for calculation of total amounts of lipid within the assessed lipid classes. A weighted calibration curve (1/Y) was used for TG and FAs, due to significant heteroscedasticity of the data^38^.

All analyses were conducted in a single randomized batch with alternation between positive and negative electrospray ionization. Calibration standards (n = 3) were analyzed prior to the first sample in the sequence and 8 blanks were evenly distributed in the batch.

### Investigation of extracellular matrix integrity

The evaluation of the ECM integrity after treatment was approached by (1) the degree of tissue dehydration expressed as water retention (%; mg of retained water per 100 mg of water content in fresh tissue), (2) staining of elastin and collagen fibers of treated tissue, using the Modified Verhoeff Van Gieson Elastic Staining^39^_,_ (3) transmission electron microscopy and (4) length-tension studies.

#### Transmission electron microscopy (TEM)

Biopsies (2 mm sections) were taken out from four different paraffin blocks containing samples of native, detergent treated, pressurized CO_2_-EtOH-H_2_O treated or pressurized CO_2_-limonene treated. The samples had previously been sectioned for H&E staining and all had been treated with benzonase nuclease, except for the native control. The biopsy was dewaxed in xylene, washed in ethanol, stained with 0.05% methylene blue in ethanol, rinsed in ethanol, acetone, followed by 1:1 mixture of Polybed-acetone and finally embedded in pure Polybed 812. The polymerised block was sectioned with a Leica UC7 ultramicrotome (Leica Microsystems GmbH, Wetzlar, Germany) and sections were mounted on a pioloform coated copper Maxtaform H5 grid. The section was contrasted with 4% Uranyl acetate followed with 1% lead citrate. Images of the samples were analysed in a Tecnai BioTWIN transmission electron microscope (FEI Company, OR, USA) at two different magnifications.

#### Wire myography experiments for length-tension studies

Length-tension studies were carried out using wire myography. The samples studied were 2 mm long segments of porcine pulmonary artery either non-treated (native) or previously submitted to pressurized CO_2_-limonene, pressurized CO_2_-EtOH-H_2_O and detergent. The pressurized treatment corresponded to the central points in the experimental design. The samples were tied to pins in three Myograph Systems (610 M and 620 M from Danish Myotechnology a/s, Aarhus, Denmark)^40^ using silk thread (6–0). The temperature inside the myograph chambers was maintained at 37 °C. Zero basal tension was ascertained in Hank’s Balanced Salt Solution (HBSS, Sigma-Aldrich). Following equilibration, preparations were stretched in pre-specified steps and force was measured after 3 min. This procedure was repeated 10 times to obtain length-tension curves for each individual sample. Length and dry weight of each sample was used to obtain an approximation of the cross-sectional area that was used to normalize force.

### Statistical and chemometric analysis

The full factorial designs were evaluated using multi-linear regression in MODDE 10.1 (Sartorius Stedim Biotech). Principal component analysis (PCA) was performed in SIMCA-P 12.0.1 (Sartorius Stedim Biotech). Differences between groups were evaluated by analysis of variance (ANOVA), with Tukeys test *post hoc*, and precision was estimated using an F-test. Significance was defined as p < 0.05. In this work, the applicability of scCO_2_ and pressurized CO_2_-cosolvent fluids to decellularize porcine pulmonary arteries was studied.

## Results and Discussion

Bio-scaffolds have been produced from animals and used for transplantations in humans^11^. A prerequisite for such an approach is that all genetic material and lipids can be removed, while maintaining the integrity of the ECM fibers^17,21,30^. In this work, the applicability of scCO_2_ and pressurized CO_2_-organic solvent mixtures for decellularization of porcine pulmonary arteries was studied.

### Lipid species in pulmonary arteries

First, we examined which lipid classes were most abundant in untreated tissue using UHPLC/QTOF-MS. In agreement with previous studies, we found high levels of several lipid species within triacylglycerides (TGs; [M+NH_4_]^+^), phosphatidylcholines (PCs; [M+H]^+^), sphingomyelins (SMs; [M+H]^+^), and fatty acids (FAs; [M − H]^−^)^34^, and relatively low levels of cholesteryl ester (CEs; [M+Na]^+^), monoacylglycerides (MGs; [M+H]^+^), phosphatidylethanolamines (PEs; [M+H]^+^) (Supplementary Table S1). TG 52:1, TG 52:2, PC 34:1, PC 38:4, SM 34:1, FA 16:0, FA 18:0 and FA 18:1 (Supplementary Fig. S2) were the most abundant lipid species, which is in line with previously published results^34^. The total content of TG, PC, SM and FA was consequently used to quantify residual lipids in treated tissue.

### Delipidation of pulmonary arteries by pressurized CO_2_

Next, we examined the effects of temperature, time and pressure on tissue delipidation, using a design of experiments (DoE) approach. However, we could not detect any significant effects of these parameters on any of the response variables with either of the solvent combinations. Hence, as TG, PC, SM, FA, and total extractable lipid residues did not differ between conditions, we pooled data for the different solvent combinations to gain power in our further analyses.

Subsequently, we compared total lipid residues in treated and untreated tissue, as determined by gravimetric analysis. Pressurized CO_2_-EtOH-H_2_O treatment efficiently reduced tissue lipid levels (p < 0.01) (see Supplementary Fig. S3). Unexpectedly, samples submitted to both scCO_2_ and pressurized CO_2_-limonene showed higher levels of residual lipids than untreated samples (p < 0.01) (Supplementary Fig. S3). Since no limonene was detected during mass spectrometric analysis (the intensity of limonene adducts were below respective spectrometry base lines), these results are not due to residual limonene being extracted in the dichloromethane fraction. An interpretation is that the treatment with scCO_2_ and pressurized CO_2_-limonene did not lead to a significant removal of lipids but it improved accessibility of lipids in subsequent extraction by dichloromethane.

**Figure 3:**
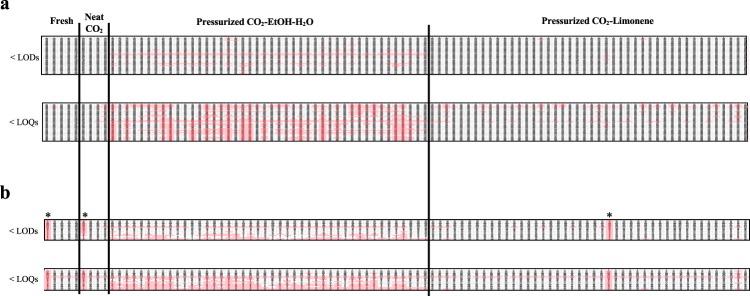
Maps of lipid species presence in untreated tissue (fresh) and samples subjected to neat scCO_2_, pressurized CO_2_-EtOH-H_2_O and pressurized CO_2_-limonene treatment for (**a**) data generated in positive electrospray ionization and (**b**) negative electrospray ionization modes. Columns represent samples clustered by treatment (at different temperatures, pressures and times) and lines correspond to lipid species sorted by class, from non-polar to polar lipids, as shown in Supplementary Table S1. Pink boxes indicate that the respective lipid specie was reduced to levels below its respective LOD (upper panel) and LOQ (lower panel). Boxes from lipids present in enough amount to be detected and/or quantified are not colored. *Outliers identified by PCA.

To generate a more comprehensive depiction of the delipidation process, we studied in detail the lipid profiles generated by mass spectrometric analysis. TGs were partially reduced for all treatments and were found at detectable levels in all samples. All PCs and SMs were also found at detectable levels in all samples, except for PC 34:0 and PC 32:1 which were undetectable after pressurized CO_2_-EtOH-H_2_O treatment, and SM 41:1 which was absent in most samples treated with this solvent combination (Fig. 3a). Overall, pressurized CO_2_-EtOH-H_2_O treatment was found to be the most efficient solvent combination for delipidation, resulting in a majority of TG, PC and SM lipid species to be reduced to levels below their respective LOQs. Therefore, concentrations of no quantifiable lipids species were ranged from 0.027, 0.919 and 1.253 ppm (i.e. TG 52:0, PC(O-34:1)/PC(P-34:0), SM 40:0) to 0.101, 4.088 and 2.381 ppm (i.e. TG 54:3, PC 32:1, SM 34:1) for TGs, PCs and SMs respectively.

Pressurized CO_2_-limonene, on the other hand, mainly reduced levels of TG 48:2 (Fig. 3a).

Pressurized CO_2_-EtOH-H_2_O was also found to be the most efficient solvent combination to reduce levels of FAs (Fig. 3b). FAs 18:1, 22:3, 22:5 and 22:6 were all absent. In addition, all long- and very long-chained FAs (C > 18), regardless their degree of unsaturation, showed levels below their respective LOQs (Fig. 3b, Supplementary Table S1).

Hence, the choice of cosolvent exerted a much more dramatic influence on PA delipidation, as compared to pressure, temperature and extraction time (Fig. 3). The extensive delipidation observed for pressurized CO_2_-EtOH-H_2_O, compared to scCO_2_ and pressurized CO_2_-limonene, are in line with results obtained for porcine retina^32^. The relative permittivity of CO_2_ is low, which makes it appropriate to dissolve mostly non-polar compounds of low molecular weight^41^. The static relative permittivity, and therefore the polarizability, of scCO_2_ can be increased with the addition of cosolvents. As an example, supercritical CO_2_-cosolvent fluids have been effective in the extraction of edible lipids like triacylglycerols and fatty acids^42^. However, there is no evidence that the addition of such small amounts of cosolvent is enough to dissolve more polar lipids, like the ones known to be present in pulmonary artery^34^. As an alternative, higher amounts of cosolvents than what is soluble in supercritical CO_2_ can be used. Under controlled conditions of pressure and temperature, this leads to a one-phase pressurized CO_2_-cosolvent fluid (see Supplementary Fig. S4), however not in the supercritical regime. Such fluids are rarely studied^31^, but they offer an even higher range of polarizability than supercritical mixtures, expanding the type of extractable lipids^32^. Mass transfer properties of such pressurized fluids are also modified with respect to the neat cosolvent, in favor of better extraction power. Furthermore, a one-phase pressurized CO_2_-cosolvent fluid prevents direct contact between the tissue and the liquid organic solvent, which would otherwise cause cytotoxic effects. By selecting ethanol as cosolvent in the pressurized fluid, we are increasing the amount of hydrogen-bonding interactions that can be created between solvent and solute. This results in higher solubilization of the polar lipids present in pulmonary arteries. In contrast to ethanol, limonene is not a polar molecule, but it is generally used to dissolve lipids in industrial applications. In the case of limonene as cosolvent, dispersion forces become stronger than for neat CO_2_, but these intermolecular interactions are still not strong enough to achieve full lipid removal of the less polar lipids (i.e. TGs) nor to dissolve the most polar lipid classes.

**Figure 4:**
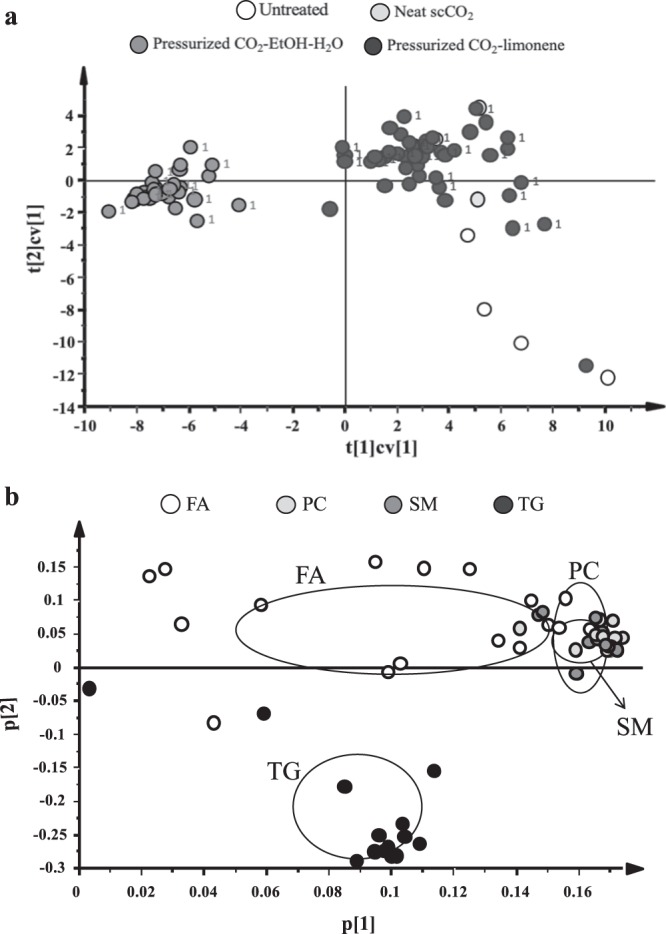
Principal component analysis calculated for lipid data from untreated, scCO_2_, pressurized CO_2_-EtOH-H_2_O and pressurized CO_2_-limonene treated porcine pulmonary arteries. (**a**) Cross-validated score-scatter plot colored by treatment. The number 1 next to the data point indicates samples selected for tissue staining. (**b**) Loading plot showing how levels of lipid species impact on the clustering observed in the score-scatter plot. Circles indicate the average ± standard deviation of the different lipid classes.

A PCA was calculated to visualize the impact of treatments on lipid profiles (described variation, R^2^ = 0.83; predictive ability Q^2^ = 0.76) (Fig. 4). The score scatter plot (Fig. 4a) revealed a clear separation between the untreated and treated PA along principal component (PC) 2, and a separation of CO_2_-EtOH-H_2_O treated PA from PA treated with CO_2_-limonene or neat scCO_2_ along PC1. The loading plot (Fig. 4b), revealed that differences in TG levels did not affect clustering as much as the other species, possibly because all treatments were partially effective at removing TGs from the tissue. The biggest impact on the clustering was due to the more polar lipids, with the biggest difference most clearly observed for PCs and SMs. CO_2_-EtOH-H_2_O was more efficient than the other conditions in extracting polar lipids from the tissue, which corresponds well with its differentiated position along PC1 in Fig. 4a. These results are in agreement with the discussion above, based on the polarizability of the solvent mixtures and the type of intermolecular interactions present.

**Figure 5:**
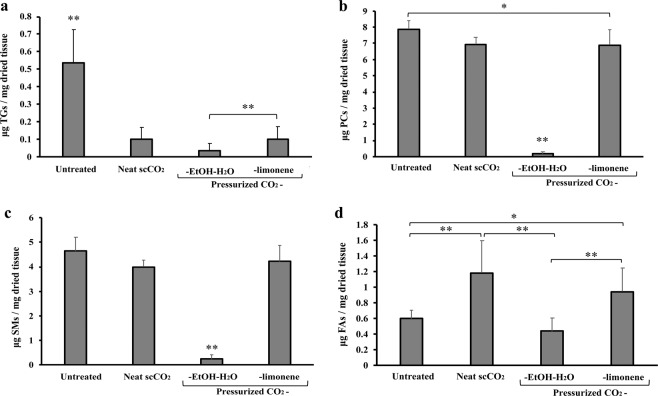
Representation of total (**a**) TG, (**b**) PC, (**c**) SM and (**d**) FA content (μg lipid/mg dried tissue) of untreated tissue and tissue subjected to neat scCO_2_, pressurized CO_2_-EtOH-H_2_O and pressurized CO_2_-limonene treatment. Data are presented as mean ± standard deviation for n = 5 (untreated and neat scCO_2_) and n = 42 (CO_2_ with cosolvent). Data were compared using ANOVA with Tukey’s test post hoc. *p < 0.05 and **p < 0.01.

**Figure 6:**
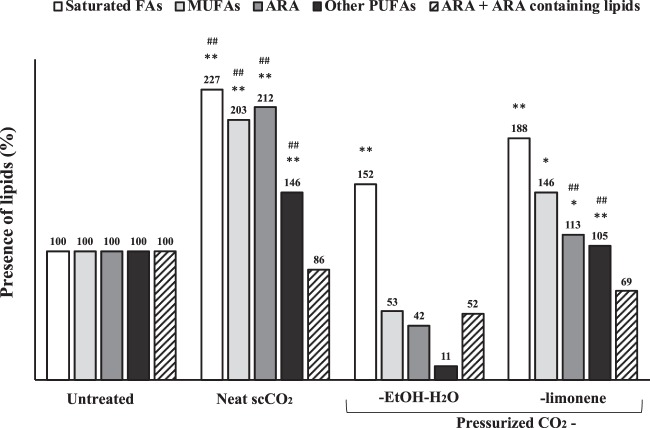
Presence of fatty acids (%) in tissues after treatment. ARA, arachidonic acid. Untreated samples expressed as 100%. * And ** denote significant differences, p < 0.05 and p < 0.01 respectively, compared with their respective fatty acids group of the untreated samples. ^#^ And ^##^ denote significant differences, p < 0.05 and p < 0.01 respectively, compared with their respective fatty acids group of the pressurized CO_2_-EtOH-H_2_O samples.

In line with the results from the PCA, univariate analysis of the lipid data revealed pressurized CO_2_-EtOH-H_2_O to be the most efficient of the tested methods for PA delipidation (Fig. 5a–c). Levels of TGs were reduced by all delipidation methods (p < 0.01), with CO_2_-EtOH-H_2_O treatment decreasing TG by 93.2% (p < 0.01) (Fig. 5a). This reduction was more significant than the reduction observed with CO_2_-limonene (81.0%). The correspondence between TG removal rates observed for scCO_2_ and pressurized CO_2_-limonene is likely governed by the similar low polarity of these fluids. Levels of PCs and SMs differed more dramatically between treatment conditions and were mainly reduced after pressurized CO_2_-EtOH-H_2_O treatment (97.8% and 94.6%, respectively; p < 0.01). Unexpectedly, the total FA content was found to increase after treatment with neat scCO_2_ (p < 0.01) and pressurized CO_2_-limonene (p < 0.05) (Fig. 5d), in line with the unexpected results from the gravimetric analysis. Similar trends have been observed for other tissue treated with pressurized CO_2_-based fluids^32^. The reason for this observation may be a result of the pressurized CO_2_ (at both supercritical or pressurized state) impacting on FA availability and facilitating subsequent extraction with dichloromethane. This effect is also observed for saturated FAs in samples treated with pressurized CO_2_-EtOH-H_2_O. The fact that we can appreciate this effect is due to that saturated FAs were not removed by the pressurized treatment to the same extent as other FAs (Fig. 6).

Pressurized CO_2_-EtOH-H_2_O was found to more efficiently remove fatty acids, as compared to neat scCO_2_ and pressurized CO_2_-limonene, presumably due to the possibility to form hydrogen bondings with the carboxylic acid moieties and OH^…^π−bonds with carbon-carbon double bonds^43^. The type of FA that remains in the tissue may affect the success of a subsequent recellularization. Notably, monounsaturated fatty acids (MUFAs) and polyunsaturated fatty acids (PUFAs), the most polar FA subclasses, were more efficiently removed by pressurized CO_2_-EtOH-H_2_O, as compared with the other treatments (Fig. 6). Levels of saturated FAs were more similar between conditions. Details on individual lipid species can be found in Supplementary Fig. S5. These are very promising results, considering that unsaturated FAs have been reported to impair proper cell adhesion, whereas the saturated FA stearic acid (FA 18:0) has been shown to induce cell adhesion^14^.

Notably, total levels of the pro-inflammatory fatty acid arachidonic acid (FA 20:4)^15^ and arachidonic acid containing lipids such as TG 54:4, TG 54:5, PC 38:4 and PC 38:5^44,45^, were reduced by 48% after treatment of the tissue with pressurized CO_2_-EtOH-H_2_O, as compared to untreated samples. ScCO_2_ treatment resulted in a less effective removal of pro-inflammatory lipids reduction (13.9%) followed by pressurized CO_2_-limonene (30.9%). Whether the reduction in arachidonic acid (FA 20:4) observed after pressurized CO_2_-EtOH-H_2_O treatment can lower proinflammatory responses after transplantation remain to be examined.

### Removal of DNA from pulmonary arteries by pressurized CO_2_

Following delipidation studies, we set out to examine whether any of the treatments also removed nuclei from the tissue, as has previously been suggested^30^, and questioned^17^ for porcine aorta. We did not find any evidence of DNA removal, assessed by both dsDNA quantification and H&E staining, using any of the pressurized CO_2_ conditions tested. In the case of scCO_2_, these results were expected due to the high polarity and molecular weight of DNA. In the case of CO_2_-cosolvent mixtures, these results are in agreement with the work by Casali *et al*.^17^ but contradict that of Sawada *et al*.^30^. The former used a mixture of CO_2_-cosolvents in the supercritical state, which is not comparable to one-phase pressurized CO_2_-cosolvent fluids. The latter is more comparable to the fluid conditions used in this work, although their mixture contains higher amounts of ethanol. It is not clear at this point if this small difference in composition explains the discrepancies in results.

Considering the unmatched Hanssen Solubility Parameter (HSP values ([δ_D_, δ_P_, δ_H_]) of [19.8, 20.1, 11.2]^46^ and [15.4, 8.7, 18.8]^47^ estimated respectively for intact DNA and a CO_2_-EtOH mixture of *X*_CO2_ = 0.31 at 40 °C and 9.3 MPa, it seems unlikely that one-phase pressurized CO_2_-ethanol fluids are able to dissolve DNA. It is likewise, in the case of limonene (HSP of [17.2, 1.8, 4.3] for neat limonene).

**Figure 7:**
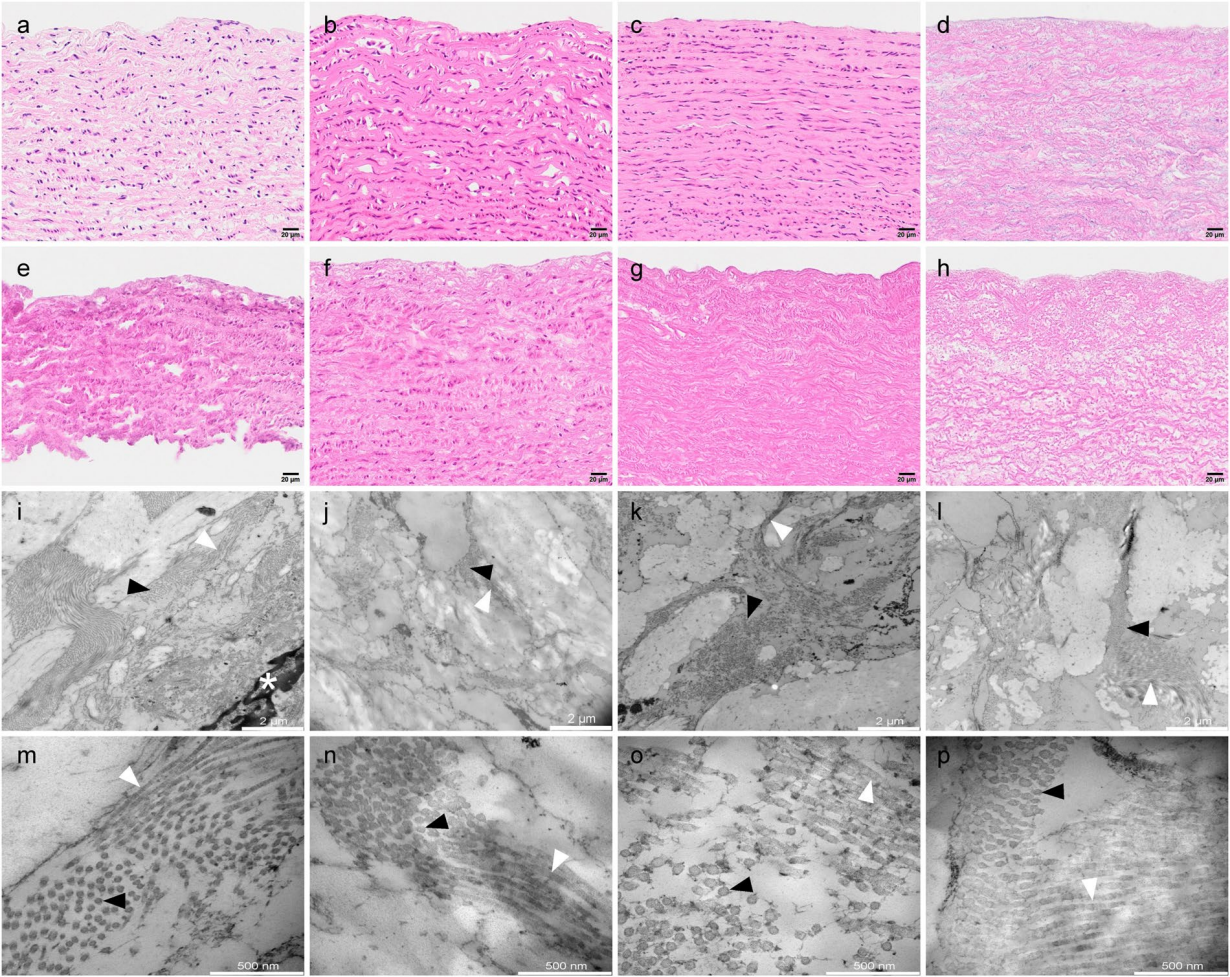
Hematoxylin and Eosin staining (**a**–**h**) and TEM images at two different magnifications (i-l and m-p, showing 2 μm and 500 nm scale bars respectively) of porcine pulmonary artery treated with pressurized CO_2_-EtOH-H_2_O and CO_2_-limonene. Representative images of (**a**,**i**,**m**) native artery, (**b**) artery treated with pressurized CO_2_-EtOH-H_2_O (30.0 MPa, 35 °C, 90 min), (**c**) artery treated with pressurized CO_2_-limonene (15.0 MPa, 40 °C, 120 min), (**d**) artery treated with a mixture of sodium deoxycholate (SDC) and Sodium dodecyl sulfate (SDS) and not subjected to pressurized CO_2_ mixtures, (**e**) artery subjected to neat scCO_2_ (30.0 MPa, 40 °C, 120 min) followed by endonuclease treatment, (**f**–**h**) same treatments as in b, c, and d with the addition of subsequent endonuclease treatment. (**j–l**) and (**n**–**p**) correspond to TEM images at low and high magnification, respectively, of the respective samples above, i.e. f, g and h. Collagen fibers in cross section (black arrowheads), longitudinally sectioned collagen fibers (white arrowheads) and cell nucleus (asterisk).

However, after nuclease treatment, one third of the pressurized CO_2_-limonene treated tissues showed a notable nuclei removal in H&E staining (see example in Fig. 7g), suggesting the absence of dsDNA. A reduction up to 93.4% of the dsDNA content compared with fresh tissues (for samples submitted to 15 MPa and 40 °C for 90 min) was achieved, resulting in an improvement of a 12.2% compared to the results from detergent-enzyme treatment. The effect of treating the sample with pressurized CO_2_-EtOH-H_2_O and neat scCO_2_ fluids prior to nuclei removal by detergent-enzyme treatment was moderate and null, respectively. There is no theory that can currently explain the promising effect of pressurized CO_2_-limonene fluid as facilitator of denaturalization of DNA by enzymes.

### Assessment of extracellular matrix integrity

Tissue dehydration is an important parameter determining the suitability of the tissue as a bio-scaffold, and a low water content has been suggested to impair the mechanical properties of the tissue^48^. However, the minimum hydration grade to preserve the functionality of a certain tissue remains unknown.

All explored fluid combinations exerted a dehydration effect. Higher water retention was observed for pressurized CO_2_-limonene and pressurized CO_2_-EtOH-H_2_O treatments, *i.e*. 16.8% and 15.0% respectively, compared to neat scCO_2_ treatment (11.4%). These results suggest that the presence of limonene and EtOH-H_2_O prevent the tissue from suffering extreme dehydration, which is in line with a previous study^17^.

We also conducted a histological examination of overall tissue structure and the integrity of elastic fibers, using a limited set of samples as indicated in Fig. 4a. This examination did reveal an overall conserved morphology with parallel aligned continuous elastic fibers without any differences between treated and untreated tissue. The spacing between elastic fibers tended to be decreased after treatment, consistent with extraction of lipids and other cellular material. The change in spacing between fibers was more pronounced for tissue samples treated with detergents (Supplementary Fig. S6).

Tissue ultrastructure was evaluated in the same samples used in the histological evaluation by reprocessing paraffin embedded samples for TEM. Tissues were stained and imaged at up to 60000 times magnification. None of the pressurized treatments exerted a distinct effect on the ECM (Fig. 7i-p). Fibrils exhibited thickness and organization comparable to the native tissue regardless of treatment with pressurized CO_2_-EtOH-H_2_O, CO_2_-limonene or detergents. Collagen fiber organization appeared intact as far as can be judged by the density and parallel appearance of the collagen fibrils. These observations were supported by length-tension studies. Our results did not show any clear differences in length-tension relationships, and thus presumably stiffness, due to sample treatments or compared to the native samples (Supplementary Fig. S7).

Decellularization and tissue integrity remains key for the production of bio-scaffolds. However, detergent and ezyme-based decellularization does not completely remove all immunogenic reactions. Previous studies have shown that decellularized xeno-transplanted tissue may still provoke immunogenic responses such as antibody formation in patients that have received decellularized porcine valves^49^. The immunogenic response appears to be induced by the ECM protein collagen VI, albumin and αGal epitopes^49,50^. These findings indicate that the ECM itself possess immunogenicity after decellularization by detergents and enzymes. Overall, the results in this work revealed that pressurized CO_2_ fluids make an impact in the removal of lipids from pulmonary artery, and in the removal of DNA when added to the battery of decellularization strategies available. Further studies are needed to evaluate if treatment with pressurized CO_2_ fluids has also a positive effect on reducing the immunogenic properties of ECM.

## Conclusion

Pressurized CO_2_-EtOH-H_2_O fluid was found to be the most efficient pressurized solvent combination to achieve lipid removal from pulmonary artery tissue. It led to the efficient removal of TGs, PCs, SMs and most FAs including pro-inflammatory lipids, and a less efficient removal of saturated FAs. Pressurized CO_2_-limonene showed a low delipidation efficiency, similar to neat scCO_2_. Further studies are needed to evaluate whether the close to complete delipidation achieved in this work results in low immune responses, cellular invasion and functional recellularization. DNA removal was more efficient with pressurized CO_2_-limonene after endonuclease treatment. Importantly, this, as well as the other tested treatments, preserved ECM integrity.

## Supplementary information

Supplementary Information.
