# Supplementary material for: Pressurized carbon dioxide as a potential tool for decellularization of pulmonary arteries for transplant purposes
Source: Sci Rep. 2020 Mar 4;10:4031. doi: 10.1038/s41598-020-60827-4 (PMC7055267; doi:10.1038/s41598-020-60827-4)
Supplement: Supplementary file 1 — Supplementary Information. [file 41598_2020_60827_MOESM1_ESM.pdf]

Supplementary material

Pressurized carbon dioxide as a potential tool for decellularization of pulmonary arteries for transplant purposes

Alicia Gil-Ramírez [a]*, Oskar Rosmark [b], Peter Spégel [a], Karl Swärd [c], Gunilla Westergren-Thorsson [b], Anna-Karin Larsson-Callerfelt [b], Irene Rodriguez-Meizoso [a]*

[a] Centre for Analysis and Synthesis, Department of Chemistry, Lund University, SE-22100, Lund, Sweden

[b] Lung Biology, Department of Experimental Medical Science, Faculty of Medicine, Lund University, SE-22184, Lund, Sweden

[c] Cellular Biomechanics, Department of Experimental Medical Science, Faculty of Medicine, Lund University, SE-22184, Lund, Sweden

*Corresponding author: aliciagilramirez@gmail.com/ irenerome@gmail.com

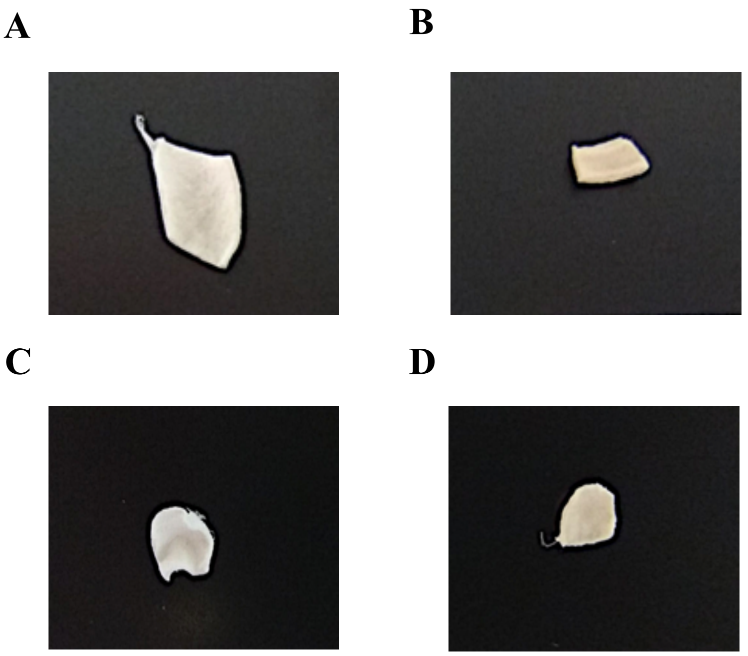

Supplementary Figure S1. Appearance of A) untreated, B) neat $scCO_2$, C) pressurized CO_2-EtOH-H_2O, and D) pressurized CO_2-limonene freeze-dried pulmonary arteries.

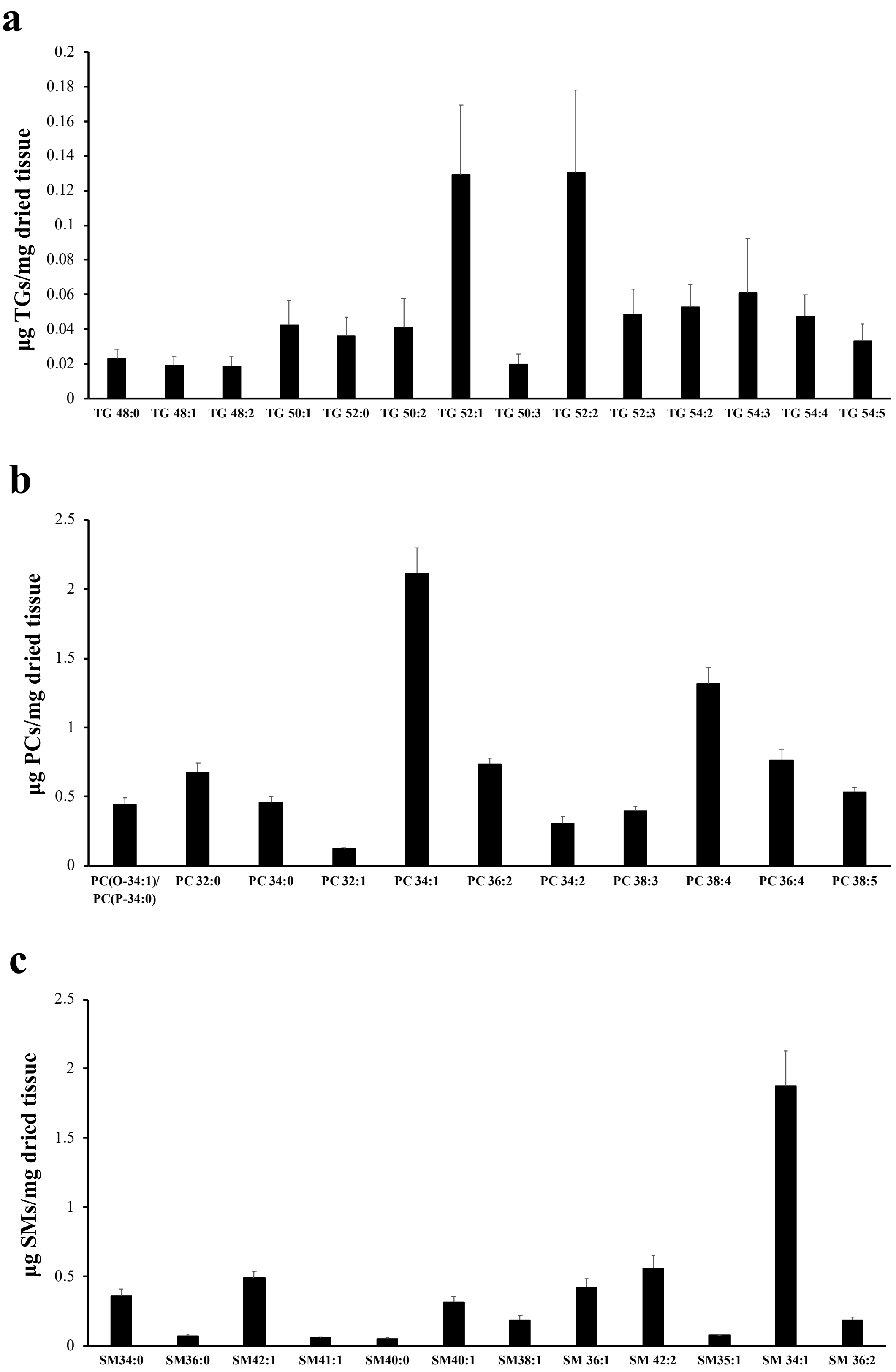

Supplementary Figure S2. Lipid species content (µg /mg dried tissue) obtained for untreated pulmonary arteries from pigs. (a) TG, triacylglycerols; (b) PC, phosphatidylcholines; (c) SM, sphingomyelins; (d) FA, fatty acids.

d

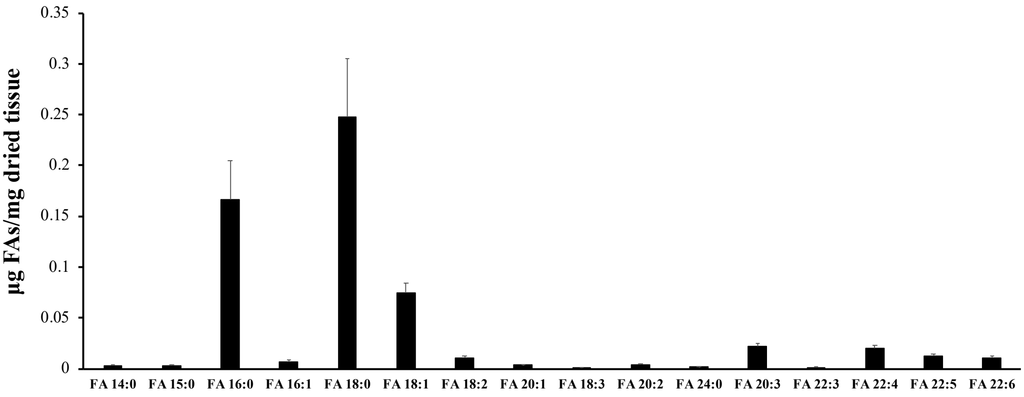

Supplementary Figure S2 (continuation). Lipid species content (µg /mg dried tissue) obtained for untreated pulmonary arteries from pigs. (a) TG, triacylglycerols; (b) PC, phosphatidylcholines; (c) SM, sphingomyelins; (d) FA, fatty acids.

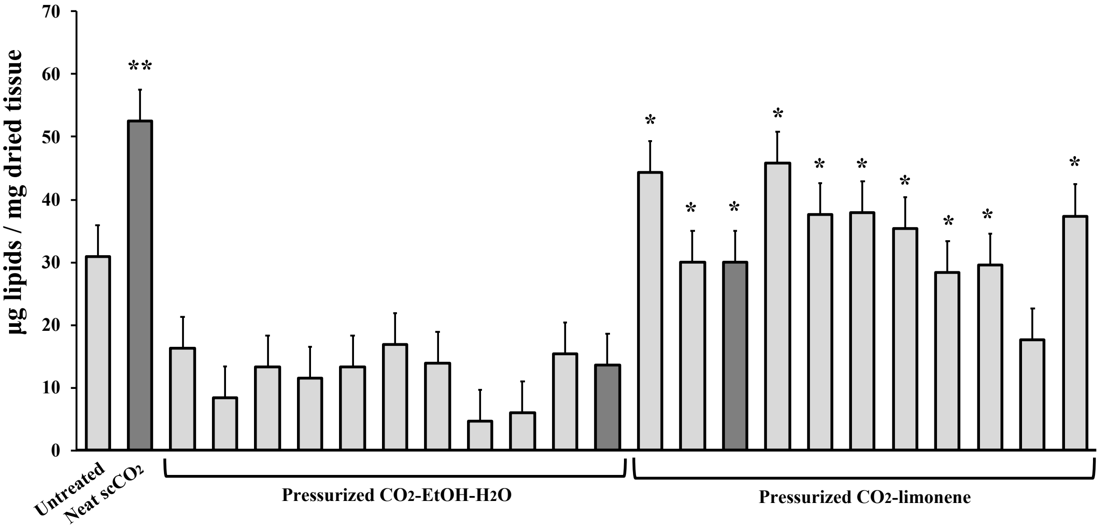

Supplementary Figure S3. Gravimetric results of residual lipid fraction (µg lipids/mg dried tissue) obtained from untreated and treated samples. Dark bars correspond to those tissues submitted to same pressure (30.0 MPa), temperature (40 °C) and time (1h). ** and * denote values statistically different from those from pressurized CO_2-EtOH-H_2O samples at a confidence level of 99% ($p<0.01$) and 95% ($p<0.05$) respectively.

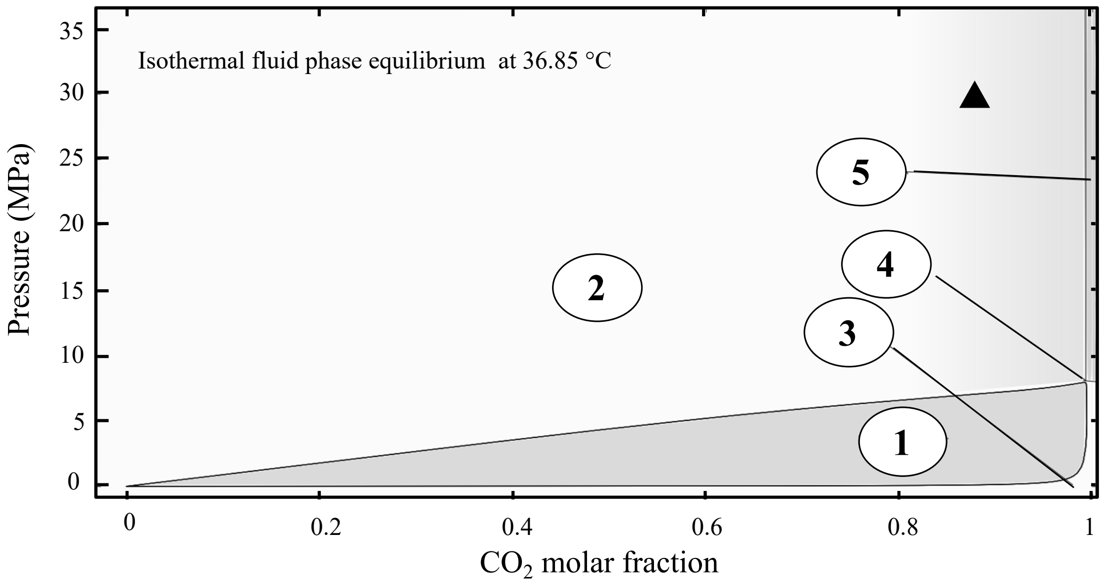

Supplementary Figure S4. Binary pressure-composition phase diagram (GPEC 3.2.1, Phasety) for the CO_2-EtOH mixture at approximately 37°C. 1. Liquid + vapour phase, 2. Liquid phase, 3. Vapour phase, 4. Critical point and 5. Supercritical region. As an example, black triangle represents the localization of a fluid similar to one used for decellularization of pulmonary arteries (i.e. pressurized CO_2-EtOH-H_2O at 30.0 MPa and 37 °C).

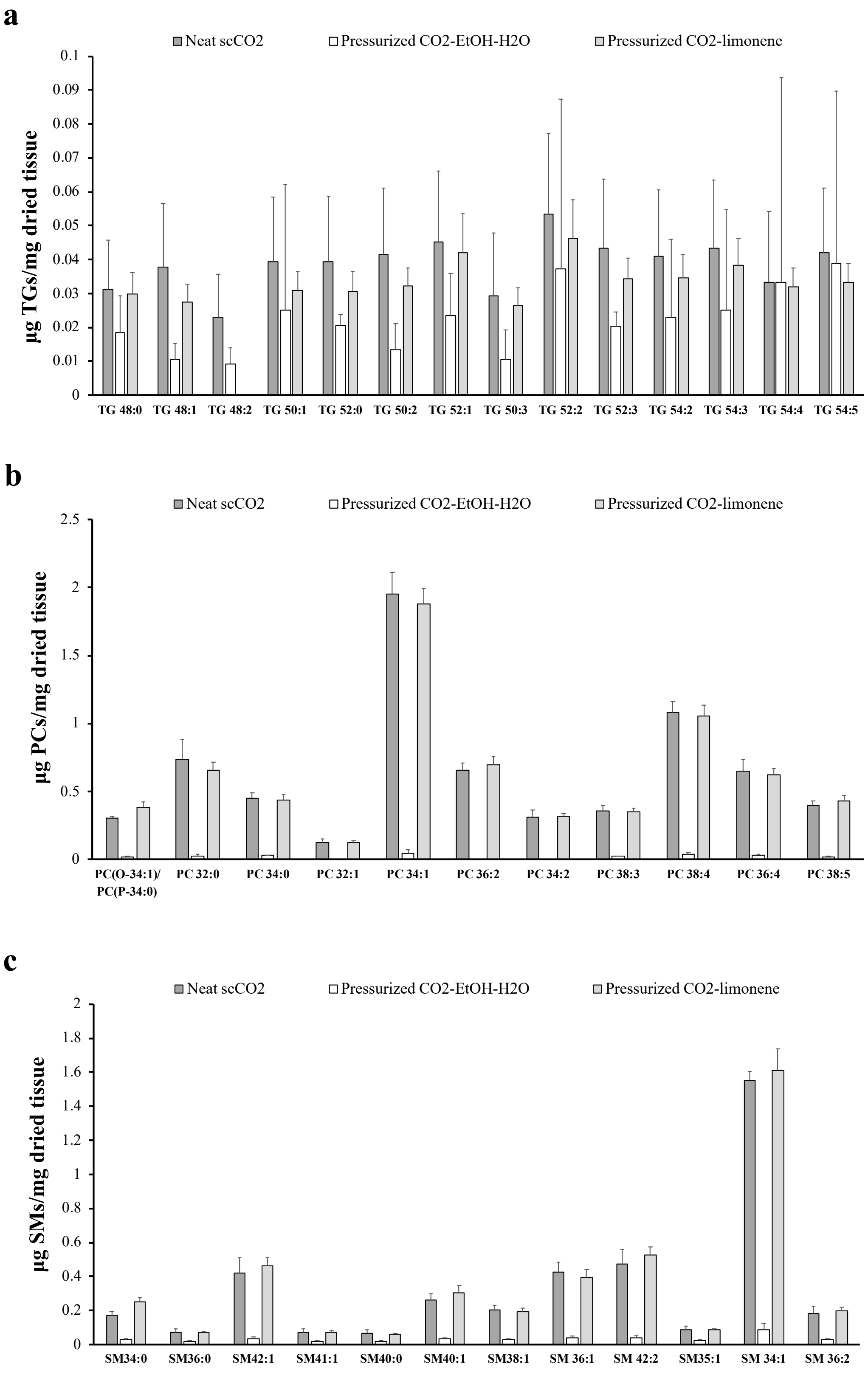

Supplementary Figure S5. Contribution of lipid species (µg lipids/mg dried tissue) to the total content of a) TGs, b) PCs, c) SMs and d) FAs lipid classes and, in consequence, to the remaining fraction of lipids after pressurized CO_2 treatments.

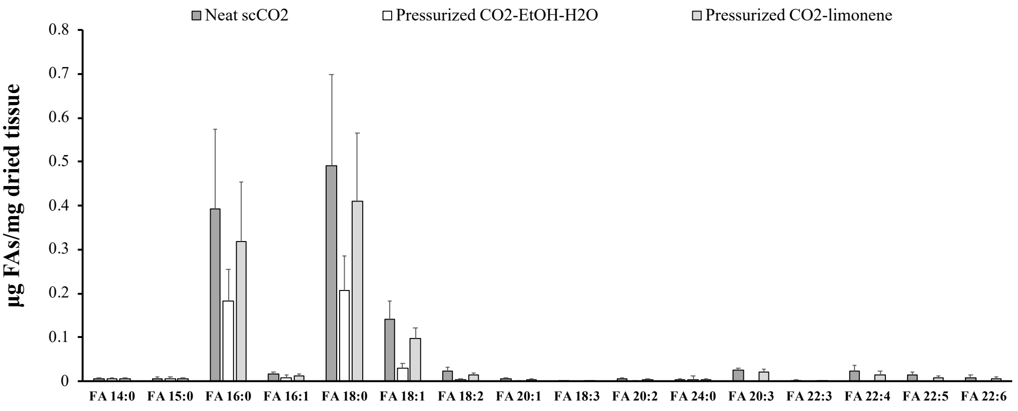

Supplementary Figure S5 (continuation). Contribution of lipid species (µg lipids/mg dried tissue) to the total content of a) TGs, b) PCs, c) SMs and d) FAs lipid classes and, in consequence, to the remaining fraction of lipids after pressurized CO_2 treatments.

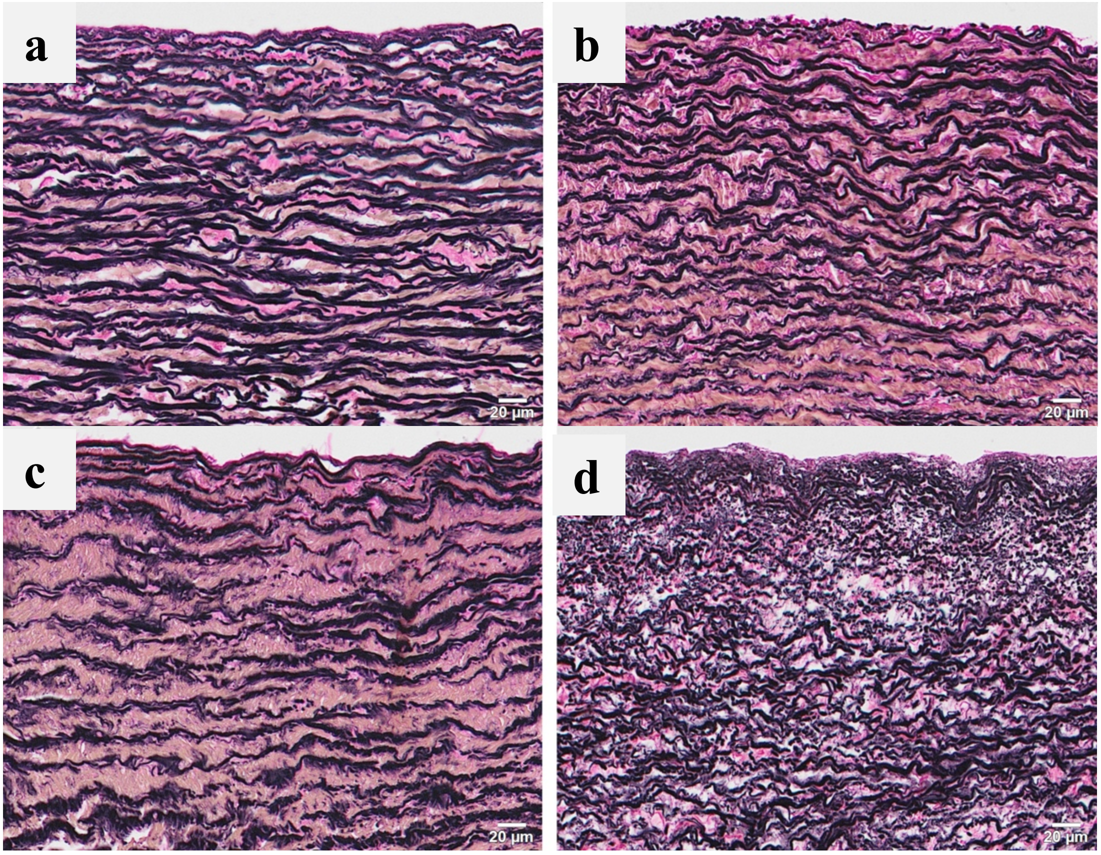

Supplementary Figure S6. Verhoeff Van Gieson staining of elastic fibers in porcine pulmonary artery. Representative images of (a) Native artery, (b) artery treated with pressurized CO_2-EtOH-H_2O,

(c) artery treated with pressurized CO_2-limonene, (d) artery treated with a mixture of sodium deoxycholate (SDC) and Sodium dodecyl sulfate (SDS) and not subjected to pressurized CO_2 mixtures. Tissue in b, c and d have all been subjected to endonuclease treatment.

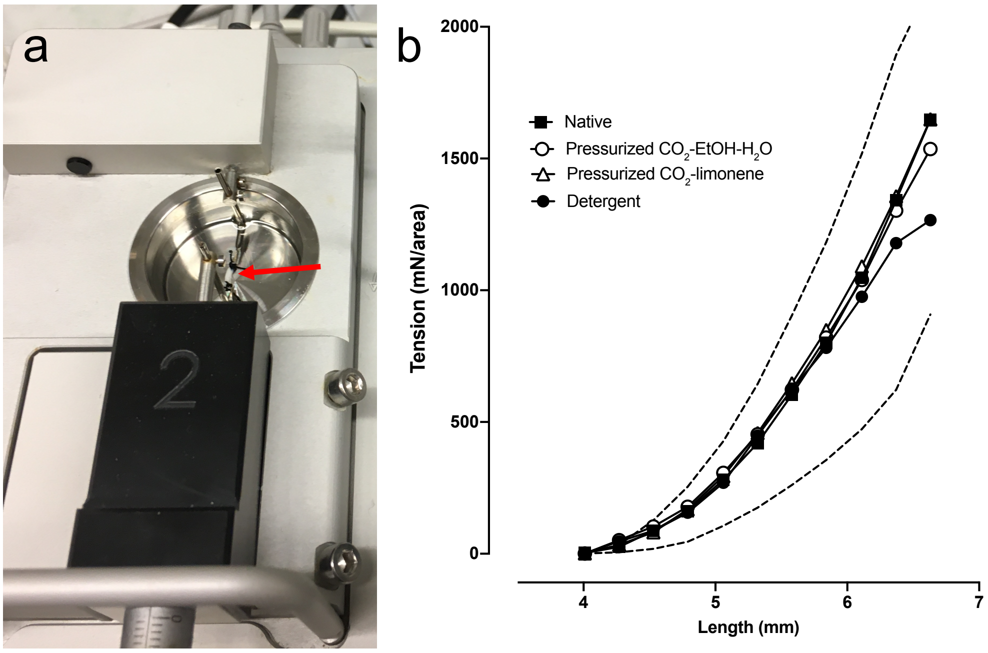

Supplementary Figure S7. (a) Picture of the wire myograph system with a 2 mm long tissue sample mounted (arrow), (b) length-tension curves of the different treatments. Data are presented as mean +/- standard error of the mean, n=3 for each group. Dashed lines indicate the standard error of the mean for all samples.

Supplementary Table S1. Lipid species found in porcine pulmonary arteries shorted by their corresponding elution in chromatography separation. Details of *m/z* experimental and theoretical per lipid specie are included as well as the *m/z* accuracy by *m/z* error calculation. LODs and LOQs, obtained by *m/z* lipid specie *vs* internal standard areas ratios obtained from IS blanks, are also shown per lipid specie. *For IS, LODs and LOQs were obtained from $CHCl_3$:MeOH (2:1, v/v) samples. IS, internal standard; Std, standard. $^1[M+NH_4]^+$, $^2[M+Na]^+$, $^3[M+H]^+$, $^4[M-H]^-$.

Lipid ID	Rt (min)	*m/z* experimental	*m/z* theoretical	*m/z* error	LOD ($\times 10^{-4}$)	LOQ ($\times 10^{-4}$)
**Triacylglycerides (TG)[1]**						
**TG 48:0**	0.698	824.7712	824.7702	1.21	5.244	17.481
**TG Std.**	0.707	824.7713	824.7719	-0.72	n.c	n.c
**TG 48:1**	0.713	822.7546	822.7545	0.12	3.186	10.619
**TG IS***	0.728	829.8010	829.8014	-0.48	0.353	1.178
**TG 48:2**	0.738	820.7395	820.7389	0.73	3.468	11.560
**TG 50:1**	0.750	850.7855	850.7858	-0.35	3.311	11.037
**TG 52:0**	0.763	880.8321	880.8328	-0.79	1.658	5.525
**TG 50:2**	0.765	848.7709	848.7702	0.82	3.589	11.963
**TG 52:1**	0.767	878.8145	878.8130	1.70	2.295	7.651
**TG 50:3**	0.783	846.7550	846.7545	0.59	1.818	6.061
**TG 52:2**	0.798	876.8026	876.8015	1.25	3.742	12.472
**TG 52:3**	0.835	874.7841	874.7806	4.00	1.786	5.952
**TG 54:2**	0.851	904.8311	904.8297	1.5	2.331	7.769
**TG 54:3**	0.914	902.8187	902.8171	1.77	6.108	20.361
**TG 54:4**	1.086	900.8036	900.8015	2.33	3.672	12.240
**TG 54:5**	1.153	898.7849	898.7858	-1.00	3.576	11.919
**Cholesteryl ester[2]**						
**CE 20:2**	3.232	699.5967	699.6050	-11.8	n.c	n.c
**Monoacylglicerides (MG)[3]**						
**MG 16:0**	3.703	353.2667	353.2668	-0.28	n.c	n.c
**MG 18:0**	3.739	381.2980	381.2981	-0.26	n.c	n.c
**MG IS***	3.780	364.3444	364.3442	0.54	n.c	n.c

**MG 18:1**	3.878	379.2824	379.2824	0	n.c	n.c
**Phosphatidylcholines (PC)[3]**						
**PC(O-34:1)/ PC(P-34:0)**	6.714	746.6058	746.6058	0	3.979	13.264
**PC 32:0**	6.752	734.5704	734.5695	1.22	15.973	53.242
**PC 34:0**	6.753	762.5981	762.6008	-3.54	79.242	264.142
**PC IS***	6.780	753.6145	753.6132	1.72	35.990	119.965
**PC 32:1**	6.781	732.5547	732.5538	1.22	249.164	830.546
**PC 34:1**	6.787	760.5859	760.5851	1.05	5.583	18.610
**PC Std.**	6.789	734.5703	734.5710	-0.9	n.c	n.c
**PC 36:2**	6.815	786.6011	786.6008	0.38	30.147	100.488
**PC 34:2**	6.823	758.5703	758.5695	1.05	13.998	46.661
**PC 38:3**	6.834	812.6155	812.6164	-1.10	11.428	38.092
**PC 38:4**	6.847	810.6011	810.6008	0.37	31.321	104.404
**PC 36:4**	6.856	782.5704	782.5695	1.15	18.642	62.139
**PC 38:5**	6.891	808.5856	808.5851	0.61	9.045	30.150
**Sphingomyelins (SM)[3] and Phosphatidylethanolamines (PE)[3]**						
**SM34:0**	7.336	705.5901	705.5923	-3.11	3.034	10.112
**SM36:0**	7.363	733.6221	733.6235	-1.90	3.599	11.996
**PE36:1**	7.371	746.5719	746.5724	-0.66	n.c	n.c
**PEO-40:5/ PEP-40:4**	7.406	780.5906	780.5909	-0.38	n.c	n.c
**PEO-38:5/ PEP-38:4**	7.406	752.5596	752.5600	-0.53	n.c	n.c
**PEO-36:5/ PEP-36:4**	7.419	724.5294	724.5300	-0.82	n.c	n.c
**SM42:1**	7.431	815.7008	815.7034	-3.18	14.666	48.887
**SM41:1**	7.436	801.6857	801.6862	-0.62	4.651	15.502
**SM40:0**	7.445	789.6846	789.6876	-3.79	1.372	4.574
**SM40:1**	7.445	787.6700	787.6703	-0.38	10.303	34.343
**SM38:1**	7.458	759.6380	759.6388	-1.05	7.192	23.972
**SM 36:1**	7.462	731.6065	731.6061	0.54	13.949	46.497
**SM 42:2**	7.466	813.6852	813.6844	0.98	27.102	90.341
**SM35:1**	7.474	717.5918	717.5923	-0.69	2.888	9.628
**SM IS***	7.486	738.6484	738.6472	1.62	44.655	148.850
**SM 34:1**	7.487	703.5759	703.5748	1.56	66.722	222.408
**SM 36:2**	7.524	729.5910	729.5905	0.68	5.358	17.860

**SM Std.**	7.535	703.5752	703.5770	-2.5	n.c	n.c
**Fatty acids (FA)[4]**						
**FA 14:0**	1.608	227.1986	227.1980	2.64	0.833	2.776
**FA 15:0**	1.651	241.2174	241.2192	-7.46	0.841	2.804
**FA 16:0**	1.749	255.2322	255.2324	-0.78	39.899	132.998
**FA 16:1**	1.837	253.2180	253.2192	-4.73	1.418	4.728
**FA Std.**	1.884	283.2635	283.2635	0	n.c	n.c
**FA 18:0**	1.885	283.2632	283.2605	9.53	24.082	80.273
**FA 18:1**	1.987	281.2485	281.2494	-3.20	24.372	81.240
**FA 18:2**	2.098	279.2308	279.2307	0.35	2.345	7.818
**FA 20:1**	2.114	309.2816	309.2813	0.96	0.146	0.486
**FA 18:3**	2.233	277.2191	277.2194	-1.08	0.232	0.773
**FA 20:2**	2.236	307.2627	307.2638	-3.57	0.205	0.684
**FA 24:0**	2.274	367.3590	367.3604	-3.81	0.698	2.327
**FA 20:3**	2.351	305.2456	305.2454	0.65	0.254	0.846
**FA IS***	2.494	314.3036	314.3040	-1.27	0.033	0.111
**FA 22:3**	2.535	333.2796	333.2793	0.90	0.065	0.216
**FA 22:4**	2.694	331.2655	331.2644	3.32	0.046	0.153
**FA 22:5**	2.874	329.2472	329.2477	-1.51	0.080	0.265
**FA 22:6**	3.089	327.2253	327.2266	-3.97	0.095	0.318
